# Unhealthy Lifestyle and Gut Dysbiosis: A Better Understanding of the Effects of Poor Diet and Nicotine on the Intestinal Microbiome

**DOI:** 10.3389/fendo.2021.667066

**Published:** 2021-06-08

**Authors:** Jason E. Martinez, Doron D. Kahana, Simran Ghuman, Haley P. Wilson, Julian Wilson, Samuel C. J. Kim, Venu Lagishetty, Jonathan P. Jacobs, Amiya P. Sinha-Hikim, Theodore C. Friedman

**Affiliations:** ^1^ Department of Internal Medicine, Charles R. Drew University of Medicine and Science, Los Angeles, CA, United States; ^2^ Department of Medicine, David Geffen School of Medicine at University of California, Los Angeles, CA, United States; ^3^ Vatche and Tamar Manoukian Division of Digestive Diseases, Department of Medicine, David Geffen School of Medicine at University of California, Los Angeles (UCLA), Los Angeles, CA, United States; ^4^ David Geffen School of Medicine at University of California, UCLA Microbiome Center, Los Angeles, CA, United States; ^5^ Division of Gastroenterology, Hepatology and Parenteral Nutrition, Veterans Administration Greater Los Angeles Healthcare System, Los Angeles, CA, United States

**Keywords:** gut microbiome, gut microbiota, gut dysbiosis, nicotine, obesity, high-fat diet, nonalcoholic fatty liver disease, e-cigarette

## Abstract

The study of the intestinal or gut microbiome is a newer field that is rapidly gaining attention. Bidirectional communication between gut microbes and the host can impact numerous biological systems regulating immunity and metabolism to either promote or negatively impact the host’s health. Habitual routines, dietary choices, socioeconomic status, education, host genetics, medical care and environmental factors can all contribute to the composition of an individual’s microbiome. A key environmental factor that may cause negative outcomes is the consumption of nicotine products. The effects of nicotine on the host can be exacerbated by poor dietary choices and together can impact the composition of the gut microbiota to promote the development of metabolic disease including non-alcoholic fatty liver disease. This review explores the contribution of nicotine, poor dietary choices and other unhealthy lifestyle factors to gut dysbiosis.

## Introduction

There is considerable variation in microbiome composition and function across individuals. This interindividual variability and plasticity of the intestinal microbiota has hindered efforts to identify a “healthy” microbiota. Diversity and microbial stability are often used as key indicators of gut health because of their inverse association with chronic disease and metabolic dysfunction (1). Reduced microbial diversity has been shown to be associated with various disease states (1). Instability of the gut microbiome can be caused by many factors, including infection, diet, exercise, sleep pattern, exposure to antibiotics, and various co-morbidities. Gut *dysbiosis* is a broad term (2) that can be defined as the imbalance of gut microbiota associated with an unhealthy outcome. Dysbiosis involves the loss of beneficial microbial input or signal and an expansion of pathogenic microbes (pathobionts). Dysbiosis is thought to trigger pro-inflammatory effects and immune dysregulation associated with various disease states, including non-alcoholic steatohepatitis (NASH) (3).

In 2014, the International Scientific Association for Probiotics and Prebiotics (ISAPP) provided evidence-based recommendations of strain-specific probiotics for defined conditions (4). The microbiome has become a rising topic for the lay public and may hold the key to information capable of reducing co-morbidities and preventing disease (1). Of note, some companies market to the public the ability to analyze the microbiome of an individual to “provide precision nutrition for metabolic disease” (5). More research is needed, especially on the cross-communication between the microbiome and host in order to understand the impact of the microbiome on human disease (3).

Often, the terms “microbiome” and “microbiota” are used interchangeably. In an effort to define the terms more precisely (6) *microbiota* will be used to define the assemblage of microorganisms present in a defined environment, whereas (7), mostly the colon, but also the upper gastrointestinal tract (8), saliva (9), middle meatus (10), bronchial wash (11), sputum (12), subgingival (13), and throat (14). *Microbiome* will refer to the entire habitat of a host region with its surrounding environmental conditions, including all microorganisms, bacteria, archaea, lower and higher eukaryotes, viruses, and their genomes (6).

As will be discussed in the body of this review, fiber-depleted diets create a condition ripe for dysbiosis. Moreover, nicotine, the exposure to which may be increasing with the rise of electronic cigarettes (e-cigarettes), has been shown to accentuate the effect of diet and potentially disrupt the microbiome and promote disease.

## Impact of Microbiota on Health

The gut is known to harbor a unique and dynamic microbiome as it is exposed to constant external stimuli, including diet, infectious agents, antibiotics and xenobiotics (15–17). Digestible and non-digestible carbohydrates, proteins, fats, polyphenols, prebiotics and probiotics can induce shifts in the microbiota and elicit effects on host immunologic and metabolic markers (18). There appears to be a close relationship between the gut microbiome, health, and diet, suggesting that improvements in health can be modulated *via* diet through the microbiota.

### Microbiome, Immune Dysfunction, and Inflammation

The microbiome serves many important functions. In healthy individuals, it confers protection from pathogenic organisms that cause infection. For example, the microbiota produce short-chain fatty acids (SCFAs) *via* fermentation of complex plant carbohydrates, providing an energy source for colonocytes to maintain full differentiation and regeneration (19). Furthermore, the microbiota synthesizes essential vitamins and amino acids, regulate fat metabolism (20, 21), and produce various small molecules that interact with the host environment. The microbiome, in turn, regulates the development of the immune system (22). For example, a healthy microbiome has an anti-inflammatory function by inhibiting histone deacetylases in regulatory T cells (T_regs_) through G-protein coupled receptors (GPCRs) (19).

The use of germ-free (GF) animals has provided evidence that specific microbiota influences the immune system differently. In 1885, Pasteur first proposed the generation of animals deprived of microorganisms to explore the relationship between microbes and their host (22). GF animals display significant defects in the development of primary (thymus and bone marrow) and secondary (lymph nodes and spleen) lymphoid organs and are associated with a decreased frequency of CD4^+^ and CD8^+^ intestinal T cell subsets. GF mice also have reduced numbers of intraepithelial lymphocytes that express the αβT cell receptor (TCR) (22, 23). Specific bacterial populations have been linked to specific T-cell effector subset development. For example, T helper 17 [Th_17_] cells, potent sources of interleukin-17 (IL-17), play a critical role in the clearance of pathogens and the maintenance of the mucosal barrier integrity (22). GF animals have an absence of Th_17_ (22) and thus, Th_17_ development is believed to be dependent on the intestinal microbiota (22). Additionally, recent studies have shown that the adhesion of certain microbes [e.g., segmented filamentous bacteria (SFB), *Citrobacter rodentium*, and *Escherichia coli* O157] is necessary to trigger a Th_17_ cell response in intestinal epithelial cells (24). This was shown by colonizing SFB in adult GF mice versus standard mouse microbiota (22, 24, 25). Colonization with SFB conferred enhanced protection compared with GF animals after infection with the bacterial pathogen citrobacter rodentium, a direct outcome of Th_17_-cell enrichment in these animal’s intestinal microbiomes (25). Interestingly, an exaggerated Th_17_ response is believed to promote autoimmune arthritis (25, 26), such that microbial signaling and host immune response requires a fine balance.

A unique bidirectional interaction between the mucosal immune system and the gut microbiota allows for the avoidance of an inappropriate immune response towards nonpathogenic microbes while suppressing pathogenic microbes (pathobionts) (27). For example, *Bifidobacterium* and lactic acid bacteria have been shown to secrete factors that hinder inflammation, presumably *via* the downregulation of interleukin-8 secretion, NF-kB dependent gene expression, and macrophage-attracting chemokine production (28). Furthermore, *Bifidobacterium* and lactic acid bacteria are associated with the upregulation of anti-inflammatory T_reg_ cell gene expression (29). Some studies suggest that microbial-derived SCFAs may be contributing to the modulation of host immune responses directly *via* G-protein-coupled receptors and epigenetic mechanisms, such as methylation activity within the promoter regions of certain genes (30, 31).

Dysbiosis, as defined above, is believed to contribute to the development of various immune-mediated conditions, including inflammatory bowel disease (IBD) (21), rheumatoid arthritis (32), type 1 diabetes mellitus (33), multiple sclerosis (34), and systemic lupus erythematosus (SLE) (35), among many others. IBD, which comprises of Crohn’s disease and ulcerative colitis, is a chronic inflammatory disease that is increasing in prevalence worldwide (36) and has been proposed to arise from an inappropriate mucosal inflammatory response to dysbiosis; this response is believed to be a result of genetic susceptibility and environmental exposure (37). IBD patients have decreased microbial diversity compared with healthy controls, alongside alterations in both composition and function of the intestinal microbiome (38–43). Jacobs and colleagues investigated if unaffected siblings and parents of individuals with IBD carry a pre-disease microbial risk state due to shared genetic and environmental factors. By studying the microbiome and metabolome of pediatric IBD patients and their unaffected first-degree relatives (43), they were able to identify a high correlation between fecal microbial and metabolomics profiles and disease status. Their research proposed that in families at risk for IBD, healthy individuals possess an intestinal microbial/metabolomic state with increased susceptibility to IBD (43). This finding highlights the ability of microbes to increase susceptibility to inflammatory disease *via* the production of bioactive metabolites, which affect immune activity and epithelial function (43).

### Microbiome and Obesity

Obesity, which confers an increased risk for numerous diseases, including hypercholesterolemia, hypertension, type 2 diabetes, cancer, non-alcoholic fatty liver disease (NAFLD), atherosclerosis, cardiovascular disease, and stroke (44, 45), is associated with gut dysbiosis (46). Intestinal microbiota influence the digestion, absorption, metabolism, and storage of ingested nutrients with profound effects on host physiology (46). Environmental and dietary factors can yield a microbiome that modulates host metabolism to promote obesity (46, 47). Advancements in studying the role of a high-fat diet (HFD) and Western diet (WD) on the microbiome has provided insights into the mechanisms of how gut dysbiosis leads to detrimental metabolic changes and why many individuals who consume a HFD or WD develop gut dysbiosis.

Studies of lean and genetically obese (*ob/ob*) mice (48, 49) and(*fa/fa*) rats (50) have revealed differences in their *metabotypes* [i.e., metabolic phenotypes (51)]. Lean and obese gut microbiomes are characterized by different representation of members of the *Bacteroidetes*, *Firmicutes* and *Actinobacteria* phyla of bacteria. One intriguing discovery that follows from these studies is the link between gut microbiomes and host energy harvest and homeostasis (52). Some individuals may harbor microbiota that are more efficient at energy harvest than others; for example, some types of bacteria may be better at processing carbohydrates than others. Other types of bacteria may be adept at manipulating host genes and metabolism in order to store energy, turn off satiety signals, or upregulate inflammatory pathways (52).

A higher baseline ratio of *Firmicutes* to *Bacteroidetes* ratio is seen in individuals with obesity, and for these subjects, a reduction in caloric intake resulted in a lower *Firmicutes* to *Bacteroidetes* ratio (53, 54). However, Magne et al. (55) reported that it was difficult to associate the *Firmicutes/Bacteroidetes* ratio with a determined health status or as a hallmark of obesity. Yet, low levels of *Bifidobacterium*, a key bacterial group, is notably linked to obesity, particularly in children (56). Furthermore, a gut microbiome that is largely dominated by *Firmicutes* showed altered methylation in gene promoters linked to obesity and cardiovascular disease (57). Some bacterial species, such as *Lactobacillus* spp., can be obesogenic or anti-obesogenic, depending on the specific strain; others have the potential to alleviate obesity-associated metabolic complications (58, 59). Part of the mechanism of action is *via* the interaction between the gut microbiota, host immunity, and gut barrier function (60, 61).

An abundance of *Akkermansia muciniphila*, has been linked to a healthy metabolic profile, with greater improvement in obesity-associated metabolic parameters (plasma triglycerides, body fat distribution, and insulin tolerance) for individuals with obesity following dietary intervention (62). These findings highlight the critical role of the gut microbiota in maintaining the metabolic integrity of the host, from energy harvest to metabolic activity. But energy intake is also balanced with energy expenditure, which segues to the topic of exercise and its impact on the microbiome.

### Exercise and the Intestinal Microbiome

Exercise has received much praise for its ability to regulate weight, insulin sensitivity, metabolic activity and contribute to overall improvement in health. There is growing evidence to support the role of exercise in regulating human intestinal microbiota (63–73). Emerging research shows that exercise training independently altered the composition and function of the gut microbiota (74–78). Matsumoto et al. originally found that 5 weeks of exercise training in animals resulted in an increased production of the short chain fatty acid butyrate, a product of the bacterial fermentation of dietary fiber by bacteria such as *Bifidobacteria* (79). Matsumoto et al. also found that exercise training in mice increased the relative abundance of butyrate-producing taxa (75, 80). Butyrate is the primary fuel for colonocytes and has been shown to increase colonic epithelial cell proliferation, regulate host immune system and gene expression, and promote the integrity of the gut barrier (74, 81, 82). Conflicting evidence still exists for exercise and the intestinal microbiome; for example, some rodent studies found that exercise reduced the ratio of *Firmicutes* to *Bacteroidetes* (77, 80, 83, 84), while others found that exercise increased the ratio (75, 76, 85). These discrepancies may be influenced by the kind and degree of exercise (e.g., in mice, voluntary wheel running or forced treadmill running), the contingencies of the diet, age of the animal, species/strain, and method of research.

In professional rugby players, Clarke et al. found that the intestinal microbiota showed an increase in *alpha* diversity (variance within the sample), with a higher relative abundance of 40 different bacterial taxa compared to lean sedentary controls (63); these athletes had a lower abundance of lactobacillus and bacteroides species than the lean sedentary group (63). Bressa et al. found that women who performed at least 3 hours of exercise per week had a greater abundance of *Faecalibacterium prausnizii, Roseburia hominis* , and *A. muciniphilia* compared to sedentary controls (64). *A. muciniphila* has been associated with lower body mass index (BMI) (62) and improved metabolic health in other studies, whereas *F. prausnitzii* and *R. hominis* are known to be butyrate producers (86). Overall, there is strong indication that exercise can benefit the intestinal microbiome by improving microbial diversity and increasing butyrate production.

### Brain-Gut-Microbiome Interactions

Communication between the gut microbiota and the brain is an important area of research. A vital bidirectional signaling system between the gastrointestinal tract and the brain helps maintain metabolic homeostasis and is regulated *via* neural (central and enteric nervous systems, CNS and ENS, respectively), immunological, and hormonal systems (87). The perturbation of these systems through external factors, such as diet or antimicrobial use, leads to alterations within stress-response mechanisms, behavior, and neurologic health (88, 89).

A well-documented, clinical association of an aberrant gut-brain axis is the manifestation of stress-related symptoms, such as anxiety or depression, which can lead to constipation or diarrhea and is sometimes diagnosed as irritable bowel syndrome (IBS) (90). In animal models, Neufield and colleagues illustrated that colonization of GF mice with specific pathogen-free microbiota decreased anxiety-like behavior in a well-validated maze model of anxiolytic action (91). The study also showed changes in the murine neurochemistry, with upregulated expression of brain-derived neurotrophic factor (BDNF) mRNA in the hippocampal dentate gyrus (91). BDNF expression is believed to be critical for supporting synaptic plasticity and neuronal differentiation and survival. Stressful exposures can reduce the expression of BDNF, thereby theoretically affecting cognitive and emotional health (92). Bistoletti and colleagues demonstrated the effect of broad-spectrum antibiotics in transiently decreasing BDNF levels and increasing anxiety behaviors in juvenile mice (93). Together, these studies provide important evidence that the brain and behavior, specifically anxiety, can be influenced by the microbiota through the gut-brain axis.

IBS pathophysiology is not fully understood, but for many patients, an element of visceral hyperalgesia is implicated and alterations in the bidirectional communication of the gut-brain axis may cause an exaggerated pain response to an otherwise normal digestive process (94). Labus and colleagues identified a correlation between brain architecture and the gut microbiota in a distinct subgroup of IBS patients, suggesting that gut microbiota and their metabolites may influence specific brain structures. The authors concluded that a microbe-gut-brain axis plays an important role in the pathophysiology of disrupted sensory processing in IBS (95).

Emerging evidence also suggests that gut microbiota play an important role in several neurological conditions, such as Parkinson’s disease (PD), Alzheimer’s disease, and multiple sclerosis (96). Several studies have observed that PD patients may have gastrointestinal disorders before displaying motor symptoms, and suggested that gut dysbiosis may be implicated, but the specific link is not clearly understood (96). Studies in rats have demonstrated that alpha-synuclein (α-syn), a protein found in neural tissue and implicated in PD, misfolds and forms clumps in neural tissue in response to gut dysbiosis (97–99). One plausible hypothesis is that the innervation of the GI tract is easily damaged (96, 97) and that ENS injury is caused by an unknown PD pathogen that may present as α-syn pathology. Several clinical studies revealed that PD patients displayed α-syn aggregates in the enteroendocrine system (96, 97) and that these aggregates are related to damage of enteric neurons and associated with GI tract dysfunction. This type of protein aggregate accumulation affects both the myenteric and submucosal plexuses of the gut in PD patients and is gradually distributed from the most distal point of the esophagus to the rectum (96). Moreover, gut dysbiosis is believed to result in upregulated inflammatory pathways that may trigger the initiation of synucleinopathy (100–102). If a dysfunctional gut contributes to PD, then there lies a strong possibility that the gut affects the brain in a host of ways, including neurological and psychiatric disease.

## Modulation of the Microbiota by Diet

### The Composition of the Intestinal Microbiome Revolves Heavily Around Diet

Dietary factors are often potent modulators of microbiota composition and function. Transient, diet-induced alterations occur independently of body weight and adiposity and are detectable in humans within 24 to 48 hours after dietary intake (103). A micronutrient-dense, high-fiber diet with sufficient water intake and high-quality protein, along with avoidance of common Western dietary components, such as saturated and trans-fat, simple sugar, refined flour, high-fructose corn syrup, and other processed foods, is believed to have a protective effect regarding intestinal dysbiosis (104).

Particularly important for the health of the microbiome are carbohydrates (CHO) that are indigestible yet metabolically available to microbes within the intestines. Termed “microbiota-accessible carbohydrates” (MACs) (105), these include fermentable fibers and non-digestible polysaccharides found in resistant starch foods, such as those originating from plants (106). Intestinal microbes contain several hundred-fold more CHO-degrading enzymes than what is produced by human enterocytes; this enables the microbes to digest MACs for their primary source of energy (46).

The importance of MACs on microbiota composition and function is documented in multiple studies. In one illustrative mouse experiment, a diet low in MACs resulted in a decrease in numerous taxa and a loss of diversity across several generations of offspring that were not recovered after reintroduction of MACs (105, 107). In humans, a low-MAC diet results in poor production of intestinal microbiota-generated SCFA, which are known to reduce inflammation through a variety of mechanisms (104). Decreased SCFA production and increased mucus foraging by the microbiota demonstrate consequences to low MAC intake (46). However, the intake of excessive calories to obtain an increase in MAC cannot be recommended due to the consequence of a caloric surplus (108). Instead, balancing caloric intake based on basal metabolic rate and total daily energy expenditure, alongside consumption of micro-nutrient dense, high-fiber, well-balanced foods, may be a better approach for optimizing gut microbial and human health (109).

A high-protein diet (HPD) is another approach for potentially negating the harmful effects of a Western diet (WD). In a study done by Wang and colleagues, rats fed a WD for 12 weeks showed an increase in body weight and fat mass. When the rats were switched to a HPD for 6 weeks, the rats had reduced fat mass without significant weight loss, a retention of muscle mass, normalized blood glucose levels, and decreased feeding after intraperitoneal injection of cholecystokinin (CCK) compared to rats with diet-induced obesity treated with CCK (110). The authors concluded that a HPD may be useful in promoting fat loss, restoring glucose homeostasis, and improving CCK sensitivity, as well as maintaining muscle mass during periods of caloric restriction. Furthermore, since the HPD-fed rats showed an enrichment of 114 operational taxonomic units (OTUs) and depletion of 188 OTUs, it was concluded that the microbiome was involved with the measured metabolic alterations. An example of the significant microbial difference is the positive association between *A. muciniphila* and *Phascolarctobacterium* with decreased fat mass in the HPD-fed rats compared to WD-fed rats (110). *A. muciniphila* was identified to correlate with fat loss and may represent a secondary mechanism for the beneficial effects of HPD (110). Furthermore, the study showed that WD-fed rats had increased cytokine expression in the hypothalamus and dorsal medulla, which was unchanged after switching to HPD (110).

Kaptan and colleagues found consumption of a low-calorie diet by adolescent rats led to an increase in microbial diversity, adult neurogenesis, BDNF levels, and improved cognition (111). Conversely, mice fed a HFD exhibited gut dysbiosis, decreased synaptic plasticity, and increased anxiety-like behaviors (112–114).

Overall, diet has an important effect on the microbiome and its ability to communicate amongst different systems in the body. The studies noted above on MACs, WD, HPD, low-calorie diets, and HFD, and their effects on the microbiome, highlight the importance of maintaining a healthy gut microbiome through various dietary interventions.

### Metabolic Health Impact of Various Diets on the Microbiome

Many diets emphasize the utility of a specific macronutrient (e.g., high-protein or low-fat) or the avoidance of a specific ingredient (e.g., dairy- or gluten-free). Several well-known diets have been studied for their ability to modulate intestinal microbiota, including WD (high animal fat/protein) (115–117), Mediterranean (MD) (high-fiber, high-monounsaturated fat, antioxidant-rich, and low in red meat) (118–120), vegetarian, vegan, and gluten-free (121–125).

The WD, which is high in animal protein and saturated fat and low in fiber, is usually low in MACs and has been shown to lead to a reduction in microbial diversity and altered functionality of the intestinal microbiota compared to control diets. Many studies document that a WD caused decreased diversity of *Bifidobacterium* and *Eubacterium species*, as well as increased *Enterobacteria* and *Bacteroides* (115–117). One mechanism by which gut microbes mediate the negative metabolic consequences of a HFD is through translocation of lipopolysaccharide (LPS), also known as endotoxin, a cell-wall component of Gram-negative bacteria. Increases in circulating LPS can occur after a high-fat meal, with exacerbated effects in individuals with obesity (126). Once in circulation, LPS elicits a potent inflammatory response *via* Toll-like receptor-4 (TLR-4) signaling, which has been implicated in the development of cardiovascular and metabolic disease (45, 127). Other functional differences include the association between the WD and an increase in the production of cancer-promoting nitrosamines (128, 129); this is likely related to the high quantity and poor quality of animal protein in the WD, especially processed meat.

The MD, largely acknowledged as a healthier diet than the WD, is characterized by intake of a beneficial fatty acid profile rich in mono- and polyunsaturated fatty acids, nondigestible fibrous plant sources and other low glycemic carbohydrates, and high levels of polyphenols, along with other antioxidants and micronutrients (119). Several studies have identified that a typical MD carries a lower risk of obesity, results in a better lipid profile, and lowers inflammation. From a microbial perspective, these characteristics were associated with increases in *Prevotella, Lactobacillus*, and *Bifidobacterium*, and decreases in *Clostridium* (130–135).

Diets enriched in fiber and fermentable, plant-based foods include vegan and vegetarian diets. These two diets were shown to promote significantly lower counts of *Bacteroides* and *Bifidobacterium species (*p < 0.001), compared to an unrestricted control diet (136). One study determined that differences in the intestinal microbiomes of subjects consuming an omnivorous diet versus subjects consuming a vegan or vegetarian diet showed significantly lower stool pH than controls (137). This is likely due to the formation of SCFA, like butyric acid, as well as lactic acid from Lactobacillus bacteria. A lower stool pH is believed to confer an element of colonization resistance against pathogens.

A fairly new diet that was initially recommended for those with celiac disease (CD) but has now gained popularity by the general population is the gluten-free diet (GFD). In patients with CD, GFD is intended to reduce the effects of an autoimmune response against deamidated gliadin (a component of gluten). However, in one study, Sanz et al. enrolled 10 healthy subjects to consume a GFD for 30 days and noted an associated decrease in beneficial populations of bacteria (*Bifidobacterium* and *Lactobacillus*), while potentially increasing unhealthy populations of bacteria; this was hypothesized to be caused by a reduced polysaccharide intake associated by GFD (121). In addition, the total number of *Enterobacteriaceae* and *E. coli* increased, theoretically increasing the risk for opportunistic pathogens (121). A different study on the effects of short-term GFD showed increases in *Clostridiaceae* and *Victivallaceae*, and reductions in *Ruminococcus bromii*, *Veillonellaceae* and *Roseburia faecis* (122). *Veillonellaceae* is considered to be a pro-inflammatory family of bacteria. The clinical consequence of GFD in non-celiac individuals is largely unknown and therefore GFD cannot be recommended for the general population based on the available data.

Intermittent fasting, another recently popular diet, led to changes in the microbiome as well as improvement in metabolic parameters (138). Eight weeks of intermittent fasting revealed that the community structure of the intestinal microbiota was not significantly changed overall, but there were changes in the abundance of *Ruminococcaceae* at the family level and *Roseburia* at the genus level. This was accompanied by an increased production of SCFA, decreased circulating levels of lipopolysaccharide (LPS) and inflammatory cytokines, ameliorated markers of oxidative stress, improved vasodilatory parameters, and reduced subject body fat mass (138). There is great need for further research on the health benefits of fasting and the role it plays in autophagy and cellular regeneration, especially in the liver.

## Effect of Nicotine on Microbiome and Interactions With Diet

Smoking cigarettes has an impact on gut health, including changes in the microbiome that can affect overall health. Nicotine, the psychoactive component of tobacco, binds to nicotinic acetylcholine receptors (nAChR), such as the *α*4/*β*2 receptor, and low-affinity receptors, such as *α*7 in the CNS and peripheral tissues (139, 140). Nicotine, when given with a HFD, leads to hepatic and muscle steatosis that is thought to be due, at least in part, to increased abdominal fat lipolysis (141–143). In an animal study, we found that the *α*7nAChR agonist PNU-282987 protects against nicotine and HFD–induced hepatic steatosis in genetically obese mice (144). In this mouse model, smoke-exposed mice showed an alteration in colonic bacterial activity and community structure, with an increase of *Lachnospiraceae sp* (145).

In human microbiome studies, tobacco smokers displayed a higher relative abundance of *Prevotella*, lower relative abundance of *Bacteroides*, and a lower Shannon diversity (a measurement of diversity) compared to controls (146). Biedermann and colleagues described a decrease of *Bacteroides* as well as alterations in the abundance of *Alphaproteobacteria* and *Betaproteobacteria* following the cessation of smoking (147). Indeed, smoking cessation induced profound changes in the gut microbiome, with an increase of *Firmicutes* and *Actinobacteria* and a decrease of *Bacteroidetes* and *Proteobacteria* at the phylum level; smoking cessation also induced an increase in microbial diversity (148). Importantly, the intestinal microbial composition of smokers and non-smokers were different when fed identical meals to avoid the influences of dietary factors (149). Other human studies have confirmed that smoking is associated with variances not only in the intestinal microbiome, but also the upper gastrointestinal tract (8), saliva (9), middle meatus (10), bronchial wash (11), sputum (12), subgingival (13), and throat (14).

Several studies investigated the intestinal microbiomes of smokers vs. non-smokers, but these involved mostly Crohn’s disease patients or had a small sample size (148, 150, 151). Nolan-Kenney and colleagues compared the composition of intestinal microbiomes in smokers vs. non-smokers by collecting stool samples in a cross-sectional study of 249 participants selected from the Health Effects of Arsenic Longitudinal Study (HEALS) in Bangladesh (147). They examined the associations between the status and the intensity of smoking with the relative abundance and presence of individual bacterial taxon, from phylum to genus (147). In current/active smokers, they found that the relative abundance of bacterial taxa among the *Eryispelotrichi*-to-*Catenibacterium* lineage was significantly higher compared to non-smokers (147). They calculated a 1.91 odds ratio (OR) (95% confidence interval [CI] = 1.36 to 2.69) for the genus *Catenibacterium* when comparing the mean relative abundance in current smokers with that in subjects who never smoked, and a 1.89 OR (95% CI = 1.39 to 2.56) for the family *Erysipelotrichaceae*, order *Erysipelotrichale*, and class *Erysipelotrichi* (false discovery rate-adjusted p-values=0.0008 to 0.01) (147). Moreover, for each of these bacterial taxa, a nicotine/smoking dose-response association was observed, with increasing mean relative abundance of specific taxa as packs per day of cigarettes increased. In addition, the presence of *Alphaproteobacteria* was significantly greater (OR = 4.85, false discovery rate-adjusted p-values = 0.04) in current smokers vs. non-smokers (147). The data are consistent with other studies that associate smoking and its intensity with a change in the intestinal microbial composition (**Table 1**), suggesting that cigarette smoking plays a significant role in gut dysbiosis, especially as the level of tobacco exposure increases.

**Table 1:** Human research studies conducted on the effect of smoking or nicotine on the microbiome.

Study	Study Purpose	Sample Size	Findings
(146)	To evaluate the effect of electronic cigarettes (EC) or tobacco smoking in oral and gut microbiota.	n = 30 (10 EC users, 10 tobacco users, 10 controls)	Tobacco smokers had higher relative abundance of *Prevotella*, lowered *Bacteroides*, and lowered Shannon diversity. No significant differences were found in alpha diversity, beta-diversity, or taxonomic relative abundances between EC users and controls.
(147)	To compare the gut microbiome of smokers versus nonsmokers.	n = 249	Bacterial taxa along the *Erysipelotrichi*-*Catenibacterium* lineage and *Alphaproteobacteria* increased in current smokers. Each taxa exhibited dose-response associations.
(148)	To assess the changes in the intestinal microbiome associated with smoking cessation.	n = 20 (10 subjects in the experimental group; 5 continuing smoker control subjects; 5 non-smoker control subjects)	Increased abundance of *Firmicutes* and *Actinobacteria* in smokers. Decreased abundance of *Bacteroidetes* and *Proteobacteria* on phylum level in smokers. Microbial diversity increased following smoking cessation.
(149)	To identify the association between human intestinal microbiota (HIM) and smoking habits *via* data mining analysis.	n = 92	Decision tree was successfully able to identify smokers and non-smokers using operational taxonomic units (OTUs) for analysis. Related OTUs were all found to be uncultured bacteria.
(8)	To assess the relationship between tobacco use and changes in the upper gastrointestinal microbiome.	n = 278 (46.8% current smokers, 12.6% former smokers, 40.6% never smokers)	Subjects were divided into current smokers and never smokers and were characterized by alpha and beta diversity of the gut microbiome. Current smokers had increased alpha (mean 42.3 species) versus never smokers (mean 38.9 species) and exhibited increased beta diversity, *Dialister invisus*, and *Megasphaera micronuciformis*.
(9)	To investigate the association of cigarette smoking with the oral microbiome.	n = 1204 (26.3% never smokers, 63.3% former smokers, 10.4% current smokers)	Current smokers had decreased *Proteobacteria* (4.6%) compared with never smokers (11.7%) at class, genus and OTU levels No difference in *Proteobacteria* was found between former and never smokers. Reduced genera *Capnocytophaga*, *Peptostreptococcus* and *Leptotrichia* in current smokers compared with never smokers. Functional analysis revealed these genera were related to carbohydrate, energy, and xenobiotic metabolism Increased *Atopobium* and *Streptococcus* in current smokers compared with never smokers.
(10)	To evaluate the relation between smoking history and sinonasal microbiome alterations in chronic rhinosinusitis (CRS) and non-CRS subjects.	n = 101 (70 CRS patients and 31 control subjects)	Univariate analysis demonstrated that genus-level compositions of the middle meatus microbiota are significantly associated with smoking (p= 0.04), preoperative antibiotics (p= 0.03), and purulence (p= 0.0002). Multivariable model demonstrated that CRS (p= 0.02), polyposis (p= 0.03), purulence (p= 0.0004), and use of saline rinses (p = 0.5) have significant interactions with smoking. Diverse bacterial taxa varied significantly in composition between never-smokers and current smokers, former smokers and CRS subtypes.
(11)	To examine microbiota found in the lower airway in patients with COPD, smokers without COPD and non-smokers.	n = 37 (18 adults with COPD, 8 smokers with no airways disease, and 11 healthy individuals)	In extended-culture analysis, the total load of aerobic and anaerobic bacteria between the three cohorts were similar. Culture-independent analysis showed increased *Pseudomonas*, greatest in the lower airways of patients with COPD. There was decreased alpha and beta diversity in the COPD group. *Bacteroidetes* (*Prevotella* spp) was increased in the non-COPD comparison groups. Co-occurrence bacterial taxa and putative core were observed within the lower airways.
(12)	To investigate the relation between host genetics and lifestyles with sputum microbiota compositions. Lifestyle factors considered include smoking, alcohol consumption, and physical activity.	n= 257	*Providencia* and *Bacteroides were* influenced by host genetic factors. Smoking had the strongest effect on the overall microbial community structure compared to other tested lifestyle factors. *Veillonella* and *Megasphaera* were increased in current-smokers, and increased further with the pack-year value and the Fagerstrom Test of Nicotine Dependence (FTND) score. *Haemophilus* decreased with the pack-year of smoking and the FTND score. Co-occurrence taxa influenced by host genetics were found together.
(13)	To examine the effect of smoking on the composition of the subgingival microbiome and associated risk for disease.	n = 200	Subgingival microbial profiles were different at all taxonomic levels in smokers compared to nonsmokers. Principle coordinate analysis: microbial community clustering performed based on smoking status. Smokers were characterized by a highly diverse, pathogen-rich, commensal-poor, anaerobic microbiome that closely resembles disease-associated communities.
(14)	To investigate the changes in the upper airway microbiome that result from smoking.	n > 4,000 adults.	Approximately 25,000 sequence reads were generated. Samples clustered in the first principal coordinate by whether they were smokers. (19% of variance). Similarly, samples clustered in the second principal coordinate by whether they were never smokers (17% of variance). Former smokers were distributed within and between both these clusters. Specific OTUs increased or decreased with respect to each of the two main clusters.
(150)	To assess the relation between smoking and intestinal microbiota in patients with active Crohn’s disease (CD).	n = 169 (103 subjects with active CD; 66 healthy controls; 29 smokers with CD; 8 smokers in the control group)	Multivariate analysis revealed increased *Bacteroides-Prevotella* in smokers (38.4%) compared with nonsmokers (28.1%). Healthy controls also exhibited increased *Bacteroides-Prevotella* (34.8%) compared to nonsmokers (24.1%). Pooled multivariate analysis showed patients with CD had higher *bifidobacteria*, higher *Bacteroides-Prevotella*, and *lower F. prausnitzii* (in comparison to healthy controls.
(151)	To evaluate changes in gut microbiota composition associated in smokers versus nonsmokers with active Crohn’s disease using a metagenomic approach.	n = 42 21 smoking and 21 nonsmoking patients with CD included	Decreased gut microbial gene richness (P=0.01), genus diversity (P<0.01), and species diversity (P=0.01) in smoking patients with CD compared to nonsmoking patients with CD. Decreased relative abundance of the genera *Collinsena* (P=0.02), *Enterohabdus* (P=0.02), and *Gordonibacter* (P=0.02) in smoking patients with CD compared to nonsmoking patients with CD.

### Electronic Cigarettes (E-Cigarettes) and Public Health

E-cigarette use is a public health crisis that is sweeping the United States; this epidemic involves not only adults, but also teens. E-cigarettes came to the markets in the mid-2000s and were advertised as ‘safer’ alternatives to conventional cigarettes and an effective way to stop smoking (152). However, e-cigarettes are much less regulated than traditional cigarettes, leading to extremely variable nicotine levels, with some reaching levels above combustible cigarettes (153). Many studies have shown detrimental effects of e-cigarette use including on the liver, heart and lung (144, 145, 154–161). In a mouse model, we have found that e-cigarette use is linked to cardiovascular and hepatic diseases (144, 155, 159). Our laboratory is studying the effects of e-cigarettes on mouse microbiota and we will be reporting our results in the near future.

The study by Stewart and colleagues (146) also found that tobacco smoking had a significant effect on the bacterial profiles when compared to e-cigarette users. The most significant associations were an increased relative abundance of *Prevotella* (*P*=0.006) and decreased *Bacteroides* (*P* =0.036) in the stool of tobacco smokers versus e-cigarette users. In contrast, no significant difference was found in the alpha diversity, beta-diversity (variability in community composition) or taxonomic relative abundances between e-cigarette users and controls. Therefore, the authors concluded that the use of e-cigarette users may represent a safer alternative compared to tobacco smoking but caution that their study was only done in a small cohort of smokers and e-cigarette users. Stewart and colleagues proposed a larger, multi-location cohort study with e-cigarettes and conventional cigarette users to provide insight into their effects on the microbiome.

### Nicotine Interaction with Diet in NAFLD and Obesity

NAFLD poses a significant health risk, affecting 20 to 40% of adults in the general American population and over 70% of individuals with obesity (44). Alongside obesity, nicotine is acknowledged as a risk factor for NAFLD (162, 163). There are at least three mechanisms by which smoking and/or nicotine appear to have adverse effects on the liver: toxic, immunologic, and oncogenic (164). The toxic effects include oxidative stress, which results in the activation of stellate cells, leading to fibrosis; an increase in proinflammatory cytokines (e.g., IL-1, IL-6, IL-8, TNF alpha) is a direct contributor to liver cell injury. The immunologic effects of smoking are both cell-mediated (e.g., apoptosis of lymphocytes, impaired natural killer cell activity) and humoral (i.e., suppression of antibody formation). The oncogenic effects of smoking include carcinogens found in cigarettes, such as hydrocarbons, nitrosamines, tar, and vinyl chloride that can lead to NAFLD. Tobacco consumption has also been implicated in the reduction of p53, a tumor-suppressing gene, which may be a common pathway of oncogenesis for many neoplasms (164).

In addition to the three mechanisms noted above, nicotine also appears to exacerbate obesity-induced hepatic steatosis (44) *via* gut dysbiosis and its influence on the pathogenesis of NAFLD (44, 165–167). When nicotine is combined with a HFD in mice, there is a significant increase in the levels of serum and hepatic triglyceride, as well as circulating free fatty acids (141, 143, 161). In mice, nicotine exacerbates hepatic steatosis through increased hepatocellular apoptosis and oxidative stress, as well as decreased phosphorylation (i.e., inactivation) of adenosine-5-monophosphate-activated protein kinase. This, in turn, results in the up-regulation of sterol response element-binding protein 1-c, fatty acid synthase, and activation of acetyl-coenzyme A-carboxylase, which yields further hepatic lipogenesis (44). Nicotine also increases endoplasmic reticulum (ER) stress (44) that modulates many factors, including nuclear factor 2 erythroid-related factor 2 (Nrf2), c-Jun N-terminal kinase (JNK), nuclear factor κB (NF-κB), and c/EBP homologous protein. These all contribute to the inflammatory process associated with smoking and are part of the cellular defense against oxidative stress, often resulting in cell death (44). For instance, Nrf2 serves as a master regulator of a cellular defense system against oxidative stress (168, 169) and JNK is activated in several animal models of obesity and also in patients with NASH. The activation of JNK has been demonstrated in HFD-induced hepatic steatosis in apoplipoprotein-E knockout mice (170) and nicotine plus HFD-induced hepatic steatosis in obese mice (141); the genetic deletion of JNK in animal models resulted in attenuation of fatty liver (171). NF-κB is an important transcription factor and primary regulator of inflammatory pathways. Consistent activation of NF-κB signaling has been documented in animal models of NAFLD as well as in patients with NASH (172). Thus, the data suggest that the use of nicotine-based products results in increased oxidative stress, upregulated inflammation, perturbed hepatic lipid homeostasis, apoptosis, and autophagy, which contribute to hepatic steatosis and progression to NASH (173).

Lastly, nicotine may contribute to increased gut permeability and has been implicated in poor outcomes in IBD patients (174). Miele and colleagues showed that patients with biopsy-proven NAFLD also experienced significantly greater gut permeability due to the disruption of intercellular tight junctions in the intestine compared to healthy volunteers (175). Both increased gut permeability and the prevalence of small intestinal bacterial overgrowth (SIBO), which is correlated with the severity of steatosis in NAFLD patients (175). Since smoking appears to induce profound changes in the intestinal microbiota (148, 176), we hypothesize that nicotine with HFD could compound and lead to increased intestinal permeability, LPS activation of TLRs and the inflammasome (167), induce changes in SCFAs metabolism (167), decreased choline availability, and increased trimethylamine production (167), all of which could contribute to a nicotine-derived pathway and result in the pathogenesis of NAFLD.

## Conclusion and Perspective

A healthy intestinal microbiome is dependent on a delicate balance of various microorganisms that is susceptible to external lifestyle factors, including unhealthy diet, lack of exercise, smoking and nicotine-exposure. Lifestyle modification can alter the variable portion of the microbiome. Exercise may hold numerous potential benefits for the health of the intestinal microbiome, not only through improved insulin sensitivity, weight loss, and improved cardiovascular health, but also through its impacts on the intestinal microbiota composition. Use of nicotine-based products (e-cigarettes and traditional cigarettes) leads to known health consequences, but also may be a major contributor to gut dysbiosis and increased gut permeability. More research is needed to confirm the importance of avoiding nicotine-based products to optimize gut health and lessen the risk of gut dysbiosis. Additionally, the effects of nicotine use on the gut immune system should be more closely evaluated. Moving forward, the ability of the microbiome to recover from external factors, such as nicotine and unhealthy diets, should also be evaluated. With the number of young adults and teens consuming nicotine *via* e-cigarettes on the rise, the long-term effect of nicotine has become more relevant. Effects of nicotine, either alone or in combination with the WD, on the intestinal microbiome remain to be elucidated.

## Author Contributions

JM and SG performed the literature review. JM wrote the first draft of the paper. All other authors contributed to the writing and editing of the paper. All authors contributed to the article and approved the submitted version.

## Funding

Funded by NIH grants MD012579-01, R25 DA050723, U54 MD007598, DOD CDMRP grant PR190942, VA CDA2 IK2CX001717, and California Tobacco-Related Disease Research Program (TRDRP) Community Practice-Based Research Implementation Award 28CP-0040.

## Conflict of Interest

The authors declare that the research was conducted in the absence of any commercial or financial relationships that could be construed as a potential conflict of interest.
